# From Crisis to Crowd Control. Commentary: A Crisis in Comparative Psychology: Where Have All the Undergraduates Gone?

**DOI:** 10.3389/fpsyg.2015.01729

**Published:** 2015-11-10

**Authors:** Ellen E. Furlong, Stephanie AuBuchon, Jessica Kraut, Netherland Joiner, Jennifer Knowles, Kali Lewis, Megan Win, Jack Furlong

**Affiliations:** ^1^Department of Psychology, Illinois Wesleyan UniversityBloomington, IL, USA; ^2^Department of Biology, Illinois Wesleyan UniversityBloomington, IL, USA; ^3^Department of Philosophy, Transylvania UniversityLexington, KY, USA

**Keywords:** undergraduate students, comparative psychology, teaching, research, dogs

Comparative psychology at Illinois Wesleyan University (IWU) is only 2 years old, but its successes address Abramson's (2015) question, illustrating how undergraduate students can enthusiastically engage in comparative psychology. Undergraduates are the next generation of comparative psychologists, and failure to engage them could result in failure of the field. Currently in her third year at IWU, EF's research and teaching in comparative cognition match Abramson's criteria for comparative psychology broadly, embodying the theoretical perspectives Abramson argues separate comparative psychology from comparative cognition (e.g., behaviorist, physiological, evolutionary; but see McMillan and Sturdy, 2015). Thus, we believe our comments generalize well to comparative psychology. EF and the undergraduate students contributing to this response represent various activities–two are currently in a comparative psychology course (KL, NJ), four are also conducting research (SA, JK, JK, MW).

EF began at IWU with a handful of research students. Now she consistently has 15–20, and her comparative psychology course is habitually over-enrolled. Here we will discuss three successful salients, some features of which overlap with Abramson's suggestions for improving student engagement: (1) the comparative psychology course, Experimental Research in Cognitive Psychology, (2) the Comparative Cognition Lab, and (3) a curricular innovation spanning two campuses.

To address the central question of Experimental Research in Cognitive Psychology (in papers, debates, and exams), students reach beyond a purely cognitive approach and explore the wide range of comparative psychology—evolutionary, ethological, behavioral, motivational, and cognitive. After initial introduction to evolution and approaches to comparative psychology, students read articles about a plurality of species (birds, frogs, nonhuman primates, voles, humans etc.) and their various behaviors (reproduction, foraging, navigating, socializing etc.). Lively debates and fruitful class discussions quickly emerge as students consider these topics perspectivally (evolution, behavior, cognition etc.) and explore the overarching question of human-animal continuity. Such clear and meaningful exchanges both enliven and provoke. Further, the course emphasizes how the field of comparative psychology itself engages in stimulating controversies (see Penn et al., 2008 and commentaries), enticing students to feel that they too can contribute significantly.

The most intense moments happen in the lab, where students directly confront debates around human-animal continuity through their own research. They write an argumentative essay and work in groups to reinforce their assigned position before breaking into smaller groups to design experiments addressing the overarching question. Upon approval of research proposals, EF arranges for dogs (3–4 per week) to visit campus to facilitate data collection. This lab experience “sells” the course and is largely responsible for the long enrollment wait-lists each semester. EF does not publicize the course herself; word-of-mouth about the lab experience seems to suffice. Thus, we recommend adding lab components to comparative psychology courses to promote student engagement.

The dog lab requirement, easy and inexpensive, allows students to confront comparative psychology research first-hand, which has the dual benefit of improving their research evaluation skills and igniting excitement for the research process. Faculty, staff, and students willingly bring dogs to class sessions, which can take place in nearly any empty conference room. Moreover, research supplies cost little—approximately $200–300 per semester. Given the palpable benefits of laboratory experience, such expenses are affordable by most institutions.

The Comparative Cognition lab also offers research opportunities, including working with dogs at local dog daycare facilities and researching a variety of animals in zoos. Generally such facilities welcome the attention. We have acquired lab space on campus (a former conference room, modified by installing linoleum floors and room dividers) where community volunteers and their dogs visit, cultivating congenial “town/gown” relations. We have more dogs registered than we can include, despite the lab having been open less than a year.

Because they can conduct satisfying research in the laboratory, zoos, and daycare facilities, students are prepared for graduate school in comparative psychology or other fields: they create research from start to finish, analyzing data, coding videos, and even engaging in lab management. Because so committed to their work, students have presented research at conferences (8 students at 2 conferences in 2013–2014) and earn authorship on publications (We are close to submitting our first few papers, all of which have student co-authors).

The final element of the flourishing comparative psychology enterprise engages a second liberal arts university and a second academic discipline. In May of 2015, EF team-taught a month-long interterm course, *Ape Sapiens: Wild Minds, Captive Dignity*, with a philosopher, JF (Furlong and Furlong, 2015). Students from IWU joined JF and students from Transylvania University to perform research with primates at two sites: the Louisville Zoo and the Primate Rescue Center (Nicholasville, KY). Combining a philosophical concern over primate captivity, and a comparative approach grounded in evolution and ethology, ethical questions naturally arose about how Zoos and Sanctuaries attend to cognitive capabilities of primates. This interdisciplinary course exemplifies how comparative psychology contributes to larger intellectual trends while imparting its wealth of knowledge to institutions housing nonhuman animals. In philosophy, a growing movement in the discourse about justice for nonhuman animals advises just such mooring in the lives of nonhumans—rather than in traditional anthropocentric values like “rights” or “personhood” (Nussbaum, 2006; Calarco, 2014). This rising trend makes comparative psychology more relevant to discussions of justice for nonhuman animals than ever before. Further, to Abramson's point, team-teaching this course across campuses makes comparative psychology accessible to students at universities without such programs. “Spreading the wealth” by collaborating across liberal arts campuses helps address the challenge of limited resources in such universities.

We believe ardently that comparative cognition research captures student imaginations best when embedded in the context of comparative psychology. Teaching and research framed in this way, along with thoughtful use of resources, community outreach, and foregrounding interdisciplinary payoffs respond to Abramson's challenge. We claim that we already use many of Abramson's suggestions, including emphasizing connections to human psychology, developing broad skills, and providing innovative teaching and research opportunities. We encourage our colleagues to provide similar opportunities for their students, so that we may protect the future of comparative psychology.

## Author contributions

EF and JF drafted the article based on initial written comments from SA and JKr who contributed their own ideas and synthesized commentary and ideas from NJ, JKn, KL, and MW. All authors reviewed multiple drafts of the article and agreed to the final draft.

### Conflict of interest statement

The authors declare that the research was conducted in the absence of any commercial or financial relationships that could be construed as a potential conflict of interest.
